# RUPEE: A fast and accurate purely geometric protein structure search

**DOI:** 10.1371/journal.pone.0213712

**Published:** 2019-03-15

**Authors:** Ronald Ayoub, Yugyung Lee

**Affiliations:** School of Computing and Engineering, University of Missouri at Kansas City, Kansas City, United States of America; University of Michigan, UNITED STATES

## Abstract

Given the close relationship between protein structure and function, protein structure searches have long played an established role in bioinformatics. Despite their maturity, existing protein structure searches either use simplifying assumptions or compromise between fast response times and quality of results. These limitations can prevent the easy and efficient exploration of relationships between protein structures, which is the norm in other areas of inquiry. To address these limitations we have developed RUPEE, a fast and accurate purely geometric structure search combining techniques from information retrieval and big data with a novel approach to encoding sequences of torsion angles. Comparing our results to the output of mTM, SSM, and the CATHEDRAL structural scan, it is clear that RUPEE has set a new bar for purely geometric big data approaches to protein structure searches. RUPEE in top-aligned mode produces equal or better results than the best available protein structure searches, and RUPEE in fast mode demonstrates the fastest response times coupled with high quality results. The RUPEE protein structure search is available at https://ayoubresearch.com. Code and data are available at https://github.com/rayoub/rupee.

## Introduction

Proteins represent the functional end-product within the central dogma of molecular biology [1]. As such, understanding protein structure is a central goal within structural bioinformatics. Protein structure determination, prediction, alignment, and search all serve to advance this understanding. Below, we present our approach to a fast, scalable, and purely geometric protein structure search we refer to with the acronym of *RUn Position Encoded Encodings* of residue descriptors (RUPEE).

Given a protein domain identifier, whole chain identifier or an uploaded PDB file, RUPEE can search for matches among domains defined in SCOPe 2.07 [2], CATH v4.2 [3], ECOD develop210 [4], or among whole chains defined in the PDB. RUPEE is able to search these databases using any identifier. For instance, you can search SCOPe using a CATH domain identifier.

RUPEE has two modes of operation, fast and top-aligned. Fast mode is significantly faster than all other protein structure searches discussed below but at the expense of accuracy. Despite this, we will show that the accuracy of RUPEE in fast mode is not far below that of the best available structure searches. On the other hand, the accuracy and response times of RUPEE in top-aligned mode are comparable to currently available protein structure searches that are commonly considered fast.

RUPEE stands out as not just another protein structure search, of which there are many. RUPEE is the first, to our knowledge, purely geometric protein structure search to achieve results as good as the best available protein structure searches. Being purely geometric, the kinds of matches that RUPEE returns are not biased toward a structure classification hierarchy such as SCOPe or sequence clusters such as the PDB-90. Moreover, without a dependence on pre-calculated results, it is easy to apply filters at any level of granularity to the protein structures RUPEE searches in order to better focus on structures of interest. As one example, domains not having the same fold classification as the query structure can be searched exclusively to investigate unexpected structural relationships across folds. In this regard, RUPEE makes a fundamental contribution to protein structure research that also provides a path for further research in the direction of big data representations of protein structures.

Besides our approach to protein structure search, we introduce a polar plot for torsion angles that may have wider applicability in the study of protein structure. Further, the *run position encoding* heuristic introduced below may have wider applicability to algorithms for character sequences containing long runs of repeats.

We first discuss some related work to provide a context for our approach followed by a description of our method. We end with a comparison of results against the mTM-align structure search [5], the secondary structure matching (SSM) search [6], and the CATHEDRAL structural scan [7] available at the CATH website.

## Related work

Pairwise alignment involves finding a set of spatial rotations and translations for two protein structures that minimizes a distance metric. Traditionally, the root mean squared deviation (RMSD) between *α*-carbons of aligned residues is minimized. However, the RMSD score does not factor in the distance between unaligned residues nor does it consider the percentage of aligned residues, that is, alignment *coverage*. RMSD scores also have some dependence on the length of the aligned proteins. On the other hand, the TM-score [8] takes all residues into account and normalizes for both coverage and length of the aligned proteins. For this reason, TM-score is frequently used in the assessment of protein structure alignments.

Pairwise alignments often favor accuracy over speed because the typical use case of aligning one protein structure to another does not impose tight response time requirements. On the other hand, a protein structure search can involve thousands of comparisons and accuracy is often balanced against speed. In this case, pairwise alignment is still useful for evaluating the results of a search, and this is the approach we take.

For pairwise alignment, Combinatorial Extensions (CE) [9] and FATCAT [10] are among the most popular tools, representing rigid and flexible protein alignments, respectively. CE performs a rigid alignment in order to minimize RMSD and FATCAT allows for a constrained number of twists in the protein chain in order to find a more flexible alignment before minimizing RMSD.

Besides CE and FATCAT, TM-align [11] and DALI [12] are pairwise alignment tools also in wide use, both offering their own distinct approaches to structure alignment. TM-align uses a rotation matrix designed to maximize the TM-score rather than minimizing the RMSD along with dynamic programming to find the best full-length alignment. DALI compares intramolecular distance matrices between two proteins to find a consistent set of matched submatrices that is used to align the proteins.

Of the pairwise alignment tools, CE, FATCAT, TM-align and DALI, we have found TM-align is the fastest while also providing high-quality pairwise structure alignments. It is for this reason that we use TM-align for RUPEE in top-aligned mode.

Whereas pairwise structure alignments only depend on the sequence of *α*-carbon coordinates, protein structure searches often introduce a further dependence on the sequence order of amino acids. This approach often takes the form of clustering proteins based on sequences and pre-calculating results for pairwise alignments among cluster representatives. Then, these pre-calculated results are used for filtering the number of structures used for comparisons against a query protein. The exact formula for combining the use of representatives and pre-calculated results varies from system to system. However, all systems using this approach share the same disadvantage, an indirect dependence on amino acid sequences. In the absence of a reliance on sequence representatives and pre-calculated results, and without sacrificing accuracy, response times suffer greatly, often taking upwards of an hour for queries to complete.

For protein structure searches, VAST [13] and the FATCAT server [14] are among the most popular. Nonetheless, these searches are slow in comparison to the structure searches we compare to in this paper, mTM, SSM, and CATHEDRAL, when pre-calculated results are not used. If given a known protein domain, VAST can return structural neighbors in seconds using pre-calculated results. However, if uploading a PDB file where pre-calculated results are not used, response times for VAST can exceed 30 minutes. Similarly, the FATCAT server, that does not use pre-calculated results, can take over an hour to send results for a search against PDB-90 representatives [15].

DALI also provides a heuristic structure search [16] of whole chains found in the PDB in addition to a tool for pairwise structure alignments using distance matrices as discussed above. In the case of searching, DALI first identifies matched PDB-90 representatives and then walks a pre-calculated graph of structural similarities to identify further matches in the PDB to gradually build up the set of structures similar to the query structure. DALI is slow in comparison to SSM, and mTM has shown better quality results than DALI [5].

Given the above, there remains a need for a purely geometric protein structure search. For the serendipitous exploration of relations between protein structures performed in the trenches, this search should be fast. Moreover, with a 10% yearly growth rate of solved structures deposited in the PDB [17], this search should be scalable. At a minimum, RUPEE takes a significant step in this direction as will be shown below.

## Methods

Broadly, we define a linear encoding of protein structure and convert this linear encoding into a bag of features. Min-hashing and locality sensitive hashing (LSH), techniques drawn from big data, are then applied to implement a protein structure indexing method that serves as the foundation for both RUPEE operating modes, fast and top-aligned.

Protein structure searches that use linear encodings are not unique [18–20]. The novelty of our approach lies in its remarkable performance given its simplicity. Additionally, elements of our approach can be isolated and found to be useful in their own right.

### Regions of torsion angles

Our first step towards a linear encoding of protein structure is to identify separable regions of permissible torsion angles, but first we introduce a new plot of torsion angles better suited to this effort.

Despite their utility and familiarity, Ramachandran plots [21] represent angular data using a square plot better suited for scalar data. This leads to the unwieldy arrangement where the top part of the plot is continuous with the bottom and the left is continuous with the right.

**Fig 1:**
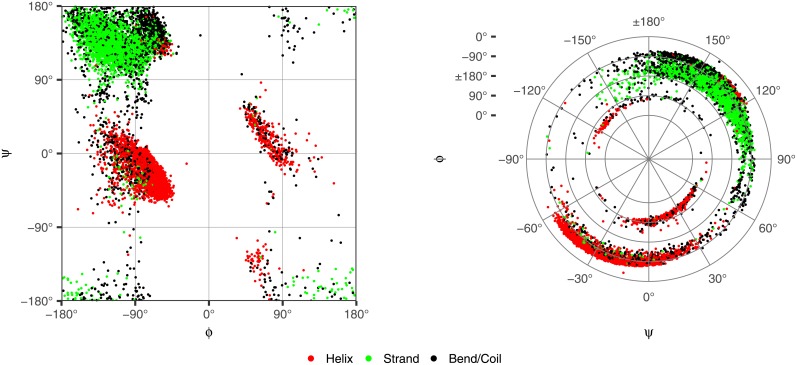
Ramachandran plot (right) and polar plot (left) of randomly sampled torsion angles.

To identify regions of torsion angles, we randomly sampled 10,000 residues from high-resolution CATH s35 representatives to account for precision and redundancy, respectively. A Ramachandran plot of the sampled torsions angles is shown in the left plot of Fig 1. As can be seen, a single cluster of residues, consisting primarily of *β*-strands, appears at all 4 corners of the Ramachandran plot.

This continuity problem was partially addressed in [22] using *wrapped* and *mirrored* plots. Both wrapped and mirrored plots take advantage of the sparsely populated areas of the Ramachandran plot at *ϕ* = 0° and *ψ* = −120°. However, with larger samples of torsion angles, the area at *ψ* = −120° becomes less sparse. The use of a polar plot resolves this elegantly by only requiring one break in continuity at *ϕ* = 0°.

In the right plot of Fig 1, we show the same torsion angles appearing in the Ramachandran plot using a polar plot. In this plot, *ϕ* corresponds to the radius *r* and *ψ* corresponds to the angle *θ* in traditional polar plots. Notice the residues appearing at the 4 corners of the Ramachandran plot now appear in one continuous region of the polar plot centered at *ϕ* = ±180° and *ψ* = ±180°.

### Linear encoding of protein structure

**Fig 2:**
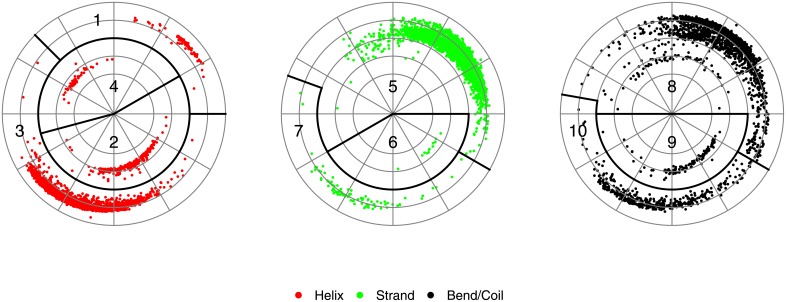
Polar plots of randomly sampled torsion angles with designated descriptors for region and DSSP code combinations.

The polar plot described above is used to define torsion angle regions for DSSP secondary structure assignments. We divide six of the eight DSSP secondary structure assignment codes defined in [23] into three groups: helices (‘G’, ‘H’, ‘I’), strands (‘E’), and bends and coils (‘S’, ‘C’). For each of the three groups, we plotted the torsion angles and identified regions into which they clustered as shown in Fig 2. The regions for helices are assigned descriptors 1 to 4, for strands 5 to 7, and for bends and coils 8 to 10. The other two DSSP secondary structure assignments codes for turns (‘T’) and bridges (‘B’), are assigned descriptors 11 and 12, respectively. For each polar plot, there are well-defined continuous regions of torsion angles that remain continuous in the plots. The only exception is found in the bends and coils plot at *ψ* = 60° between *ϕ* = −180° and *ϕ* = 0°.

**Fig 3:**
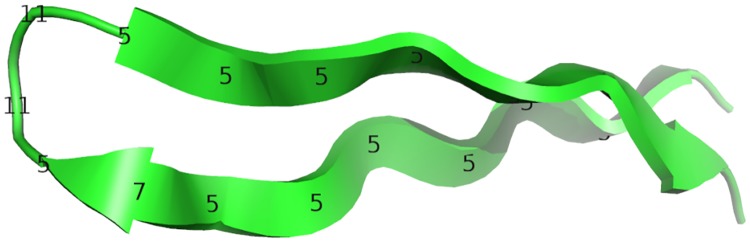
*β*-turn-*β* motif from CATH domain 1nycA00.

We use the above numeric descriptors to create a linear encoding of protein structures. For example, we apply our linear encoding to the *β*-turn-*β* motif shown in Fig 3. The corresponding sequence of residue descriptors is shown below.

[5,5,5,5,5,5,7,5,11,11,5,5,5,5,5,5] (1)

### Bag representation of protein structure

Once a linear encoding for a protein structure is obtained, it needs to be further transformed into a representation suitable for fast and scalable similarity comparisons to other structures. The processing of text documents within Information Retrieval (IR) has long been used to satisfy these requirements using bag representations. There are two distinct categories of representations for documents, syntactic and semantic, and much of the research applying IR to protein structure search has focused on the latter [24–26].

We have adapted the syntactic approach to document similarity, often referred to as shingling [27], to our linear encoding of protein structure. We transform a linear sequence of descriptors into a multiset of shingles consisting of 3 consecutive descriptors. The overlap between shingles ensures some of the order information within the original sequence is preserved in the bag.

By shingling, we obtain a multiset of ordered lists from an ordered list of numbers. As an example, the sequence in (1) is transformed into the following bag of shingles.

$$\begin{array}{c} \begin{array}{cc} \{\; & \left[5,5,5],[5,5,5],[5,5,5],[5,5,5],[5,5,7\right]\\ & \left[5,7,5],[7,5,11],[5,11,11],[11,11,5],[11,5,5\right]\\ & \left[5,5,5],[5,5,5],[5,5,5],[5,5,5]\;\right\} \end{array} \end{array}$$ (2)

Next, each shingle *s* is hashed to an integer as shown in (3). The hash function used is a simplification of the hash function used in the Rabin-Karp algorithm [28]. The prime number 13 is used as the base since it is large enough to spread the descriptor values out in hash space without collisions.

$$s_{hash}=s_{1}\times 13^{2}+s_{2}\times 13+s_{3}$$ (3)

Subsequently, the multiset in (2) becomes the following bag of integers.

$$\begin{array}{l} \{\;915,915,915,915,917,941,1259\\ \;999,2007,1929,915,915,915,915\;\} \end{array}$$ (4)

This final step completes the transformation of an ordered list of descriptors to a multiset of integers that still retains some of the order information present in the original list.

Notice in (4) the value 915, corresponding to the shingle [5, 5, 5], occurs frequently indicating the presence of *β*-strands. Since most proteins are dominated by regular secondary structure, the abundance of shingles for *β*-strands as well as the three types of helices, end up dominating comparisons. Moreover, since shingles are limited in length, this situation allows for structures with many short *β*-strands to match structures with fewer long *β*-strands. The same situation applies to helices.

To address this lack of specificity, we introduce a heuristic we call *run position encoding* (RPE), where a run is a consecutive sequence of identical descriptors. To distinguish between short and long runs, thereby increasing the specificity of the shingles, we add a factor of 10^5^ to each shingle hash as a function of the first residue’s position in a run *i*.

$$runfactor\left(i\right)=\{\begin{array}{cc} i & \text{if}\;i<\lfloor l/2\rfloor \\ l-i-1 & \text{otherwise} \end{array}$$ (5)

where *i* is zero-based and *l* is the length of the run. Multiplying by 10^5^ places the run factor as the left-most digit in the hash to avoid interference with the digits provided by the hash in (3). This placement is also convenient for visual inspection, since the run factor is isolated as the left-most digit.

The run factors for the sequence in (1) are

[0,1,2,2,1,0,0,0,0,0,0,1,2,2,1,0]. (6)

Applied to the bag of integers in (4) gives

$$\begin{array}{l} \{\;00915,10915,20915,20915,10917,00941,01259\\ \;00999,02007,01929,00915,10915,20915,20915\;\} \end{array}$$ (7)

where the leading zero run factors are shown for clarity.

This pyramidal approach preserves matches at the boundaries between secondary structure runs and loops that would not otherwise be preserved in the presence of differences in run lengths of one or more.

Now that we have a representation of a protein structure as a bag of integers, similarity for a candidate pair of structures *a* and *b* is defined as the Jaccard similarity [29] for multisets,

$$J\left(a,b\right)=\frac{\sum_{i}min\left(a_{i},b_{i}\right)}{\sum_{i}max\left(a_{i},b_{i}\right)}\text{,}$$ (8)

where *i* ranges over all possible shingle hashes *s*_*i*_ and *a*_*i*_ and *b*_*i*_ give the counts of shingle hash *s*_*i*_ in structures *a* and *b*, respectively.

In applying the Jaccard similarity to sets of shingles, the length of a shingle is chosen to balance false positives, in the case of shorter shingles, against false negatives, in the case of longer shingles. In [30], we used 4 consecutive descriptors for our shingles. The additional requirements for structural similarity we introduce in the *Operating modes* section below has the ultimate effect of reducing false positives, so this allowed us to decrease the shingle length to 3 to control better for false negatives.

### Min-hashing and LSH

In IR, the bag of shingles representation of documents is used in the near dupe clustering of documents [31]. One application of near dupe clustering is in the review stage of Electronic-Discovery [32], which is the most expensive stage in a discovery process. Often millions of documents must be examined by a staff of attorneys to make a reasonable effort at providing all documents relevant to the discovery request. Grouping documents into near dupe clusters and assigning all documents within a cluster to a single reviewer reduces duplication of effort.

In the case of near dupe clustering, each document must be compared to every other document in the collection, taking quadratic time. For this task, min-hashing [33] and locality sensitive hashing (LSH) [34] can be combined to reduce this to subquadratic time. Although we do not near dupe cluster domains, we can still leverage the techniques of min-hashing and LSH to speed up protein structure search by a large constant factor.

Min-hashing generally is used to create a fixed-length signature for a set of items by repeatedly randomly hashing the items, sorting the hashes into a list, and then selecting the minimum hash in each permuted list. If the same process is performed on a pair of sets, the key result is that the probability of matching min-hashes at the same position is equal to the Jaccard similarity of the two sets [33]. In order to approximate the Jaccard similarity for a given pair of sets, a sufficient number of min-hashes must be obtained.

In our case, the sets contain shingles for a protein structure from which we obtain 99 min-hashes for each set as described in [35]. At this stage, we have reduced our representation of protein structures from variable-sized sets of shingles to fixed-length signatures. Given the key result above, the Jaccard similarity for any pair of protein structures can now be approximated by the proportion of matching min-hashes.

We use the LSH banding technique as described in [35] to first identify candidate matches without having to compare min-hashes for every protein structure. We divide the 99 min-hashes in the signatures into contiguous subsequences (i.e. bands) of 3 min-hashes each, giving a total of 33 bands. For each band, the min-hashes it contains are combined into yet another hash. The key result of the banding technique is that if *any* band hashes are a match for a given pair of structures, we can calculate the probability that a specific Jaccard similarity threshold has been met. For 33 bands of 3 min-hashes, the probability of a band match occurring for a pair of structures having a Jaccard similarity of 60% or greater is approximately 99%. Banding allows the problem of finding similar structures to be parallelized across bands since all that is needed for a candidate match is a single band match.

Together, min-hashing and LSH provide the foundation for both operating modes of RUPEE, fast and top-aligned.

### Operating modes

RUPEE provides two modes, fast and top-aligned. Each operating mode builds on the results provided by the min-hashing and LSH system described above. When a structure search is executed in either mode, a number of concurrent tasks are executed corresponding to the 33 bands used for LSH. These concurrent tasks identify candidate matches based on a single band match; for each candidate match, we estimate the Jaccard similarity using the min-hashes. In this way, RUPEE quickly compares the query structure to every structure in the database, which in the case of ECOD is greater than 600,000 protein domains.

In fast mode, the top 8000 matches from the min-hashing and LSH initial filtering are obtained. For each match, we adjust the Jaccard similarity to account for matched shingles that are out of sequence order. For this adjustment, we calculate the exact denominator in the definition of Jaccard similarity from the original shingle sequences of the structures. As for the numerator, instead of using the total number of matched shingles, we use the length of the longest common subsequence (LCS) of the shingle sequences for each match. This LCS adjustment to the Jaccard similarity enforces the constraint that all valid shingles matches must appear in order along the length of each structure. The final step of fast mode is to sort the matches based on the adjusted Jaccard similarity scores and return the results.

Top-aligned is an additional step following fast mode that uses TM-align to identify the best pairwise alignments among the 8000 matches returned from fast mode. First, we execute TM-align on the 8000 matches obtained from fast mode using a reduced number of dynamic programming iterations in the TM-align algorithm. We sort these initial alignments by the sort criterion entered by the user, either RMSD or TM-score, and obtain the top 400 matches. Finally, we execute TM-align using the default number of dynamic programming iterations on the top 400 matches and return the sorted results based on the sort criterion.

The filter sizes of 8000 and 400 have been chosen based on quality of results and speed. We have found that increasing the size of either of these filters results in only marginal improvements in the quality of results. Given that performing pairwise alignments is the most time-consuming aspect of the RUPEE structure search, the marginal improvements gained from larger filter sizes have to be balanced against the number of pairwise alignments performed.

Top-aligned is a simple step following fast mode that establishes RUPEE fast mode as an effective filtering method that contains in its top 8000 results enough good matches to compete with the best available structure searches.

## Results

Protein structure searches can be evaluated using pairwise alignment scores (e.g. RMSD and TM-score as described above) or by comparison of results against the hierarchy of a protein structure classification database. Among protein structure classification databases for which corresponding structure searches exist, SCOPe [2] and CATH [3] are the most popular.

For our results, we have derived 3 benchmarks, scop_d360, scop_d62, and cath_d99, for pairwise evaluations to mTM, SSM, and CATHEDRAL, respectively. To avoid introducing our own bias, we were careful to derive each benchmark from an existing benchmark used in a previously published work or an existing list of protein domains provided by an independent third party. scop_d360 is derived from the d500 benchmark used in [5] filtered for domains in SCOPe 2.07 for which mTM returns 100 or more results. scoo_d360 contains domains from 262 distinct folds. Similarly, scop_d62 is derived from the d500 benchmark filtered for domains defined in SCOP 1.73 for which SSM returns 50 or more results. scop_d62 contains domains from 53 distinct folds. For all domains contained in the d500 benchmark, RUPEE returns 100 or more results. In keeping with our description of RUPEE in [30], the cath_d99 benchmark contains 99 superfamily representatives from the top 100 most diverse superfamilies defined in CATH v4.2 for which CATHEDRAL returns results in less than 12 hours.

We perform pairwise evaluations to ensure the fairness of our comparisons. First, for domain searches, SSM is working with the SCOP 1.73 database, so accordingly we operate RUPEE on SCOP 1.73 domains to ensure RUPEE does not have more domains to work with for scoring and precision evaluations. Second, mTM is updated to work with SCOPe 2.07 domain definitions but still retains domains from 2.06 that have since been redefined either through mergers or splits in 2.07. On the other hand, CATHEDRAL presents no such challenges but still requires a separate benchmark since it is working with a distinct set of domains, CATH v4.2.

All benchmark definitions can be found in S1 Benchmarks.

### Alignment normalization

The differences in approach among pairwise structure alignment tools can complicate efforts to achieve a fair comparison of protein structure searches that depend on them. Specifically, while RUPEE top-aligned and mTM both use TM-align for pairwise structure alignments, RUPEE normalizes by the average length of the aligned structures whereas mTM normalizes by the length of the query structure. Given how close the RUPEE top-aligned and mTM results shown below are with respect to scoring and precision, it is important to first examine the impact of each kind of normalization on the comparisons and also explain why we have chosen to normalize by the average length of the aligned structures for RUPEE top-aligned.

By default, TM-align normalizes by the length of the query structure, that is, the first structure passed to it. This normalization is asymmetric since higher TM-scores result when the query structure is smaller than the target structure and conversely, lower TM-scores result when the query structure is larger than the target structure. In extreme cases, high TM-scores can be achieved even when the target structure is 10 times larger than the query structure so long as the query structure can be aligned somewhere within the target structure. Since mTM uses the default asymmetric normalization provided by TM-align, this asymmetry becomes apparent in a detailed examination of mTM results.

RUPEE top-aligned uses the TM-align option of normalizing by the average length. This results in symmetric scoring where the order of the structures in the alignment has no effect on the TM-score. The first reason we use symmetric scoring in RUPEE top-aligned is to synergistically match the min-hashing and LSH layer, which filters results based on the Jaccard similarity, a symmetric similarity measure. If, on the other hand, RUPEE top-aligned normalizes by the query structure, scoring and precision are negatively effected. The second reason is that we plan to extend RUPEE to include both *contained by* and *contains* searches and do not want to favor one at the expense of the other by using asymmetric scoring.

To examine how these difference effect RUPEE top-aligned and mTM results, we gathered the maximum percentage differences in length among the results for each structure search for domains in scop_d360. We averaged these maximum percentage differences and found the average for RUPEE is 19.4% and for mTM is 90.8%. Whereas for RUPEE, these difference reflect larger and smaller query structures equally, for mTM, the larger difference always reflect a query structure smaller than the target structure. Most of the extreme cases of mTM matching a small structure to a larger structure several times its size is matching small helical fragments to larger transmembrane proteins or other proteins with helical bundles.

Given the difference in normalization between RUPEE and mTM, to be fair, we compare RUPEE scoring with mTM scoring below using TM-align normalized by the length of the query structure in addition to the average length of aligned structures.

### Scoring

**Fig 4:**
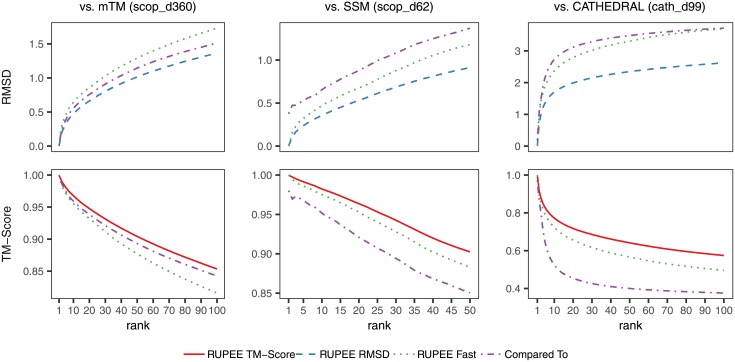
Scoring from TM-align pairwise alignments normalized by the average length of aligned structures for RUPEE fast, RUPEE top-aligned sorted by TM-score, and RUPEE top-aligned sorted by RMSD.

Fig 4 shows average cumulative values for each ranked result averaged over all searches. Both RMSD and TM-score values are shown, provided as outputs from TM-align pairwise alignments normalized by the average length of aligned structures. A TM-score above 0.5 is a good predictor for whether or not two domains are in the same fold [36]. TM-scores greater than 0.17 are considered potentially meaningful whereas TM-scores less than 0.17 are considered to be due to random alignment [8].

RUPEE fast, top-aligned sorted by RMSD, and top-aligned sorted by TM-score, perform better than SSM and CATHEDRAL. The scoring in the cath_d99 benchmark comparisons are notably lower than for the other two benchmarks. This is expected since CATHEDRAL only returns CATH s35 representatives. Likewise, for this comparison RUPEE is filtered for s35 representatives to match. Given that the cath_d99 benchmark is evaluated against representatives, there are fewer highly similar structures returned in the results.

In our evaluation, mTM faired better than SSM and CATHEDRAL. mTM also performed better than RUPEE fast, although RUPEE fast is still within 0.08 TM-score points of mTM at the 100^th^ result, which is notable considering its speed.

For both TM-score and RMSD, RUPEE top-aligned performed better than mTM. For RMSD, RUPEE top-aligned does perform better than mTM but this can most likely be attributed to the fact that mTM only sorts by TM-score. If mTM sorted by RMSD, their results likely will be improved. Nevertheless, it is worth noting that the initial min-hashing and LSH technique used by RUPEE does not explicitly bias results towards one particular measure.

**Fig 5:**
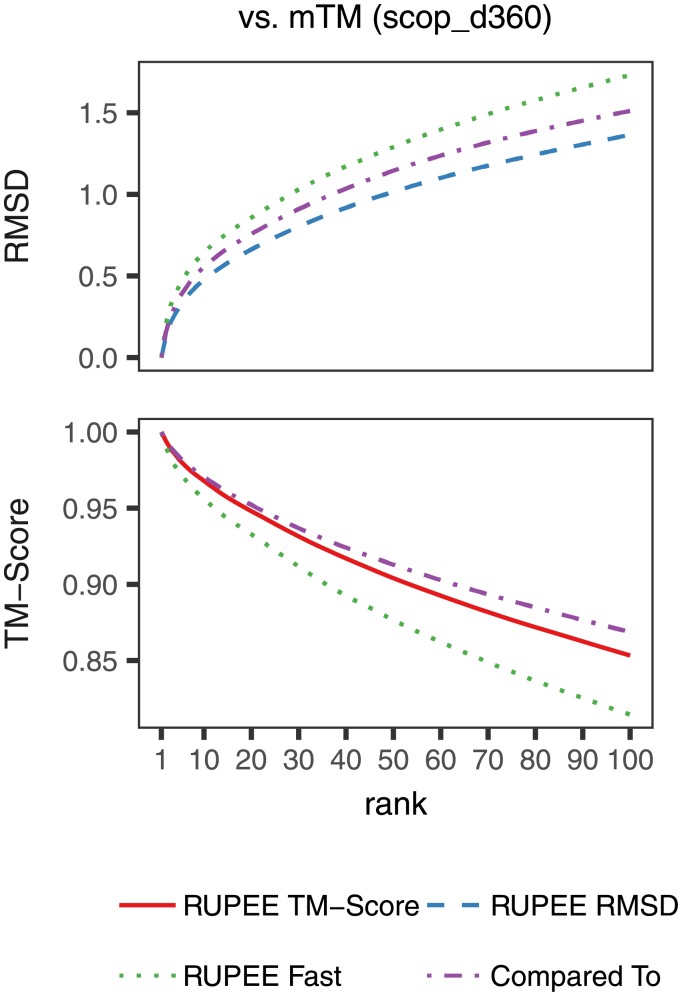
Scoring from TM-align pairwise alignments normalized by the length of the query structure for RUPEE fast, RUPEE top-aligned sorted by TM-score, and RUPEE top-aligned sorted by RMSD.

Fig 5 again shows average cumulative values for each ranked result averaged over all searches but only for RUPEE and mTM. The difference here is that instead of normalizing by the average length of the protein structures we show results for TM-align normalized by the length of the query structure. This time, mTM performs better than RUPEE top-aligned sorted by TM-score.

Taken together, Figs 4 and 5 show that the results of RUPEE and mTM are roughly equal and where they do differ is a result of the differences in normalization.

Figures similar to Fig 4 but instead using CE and FATCAT for pairwise comparisons can be found in S1 and S2 Figs, respectively. These additional figures lend further support to what has already been shown above.

### Precision

**Fig 6:**
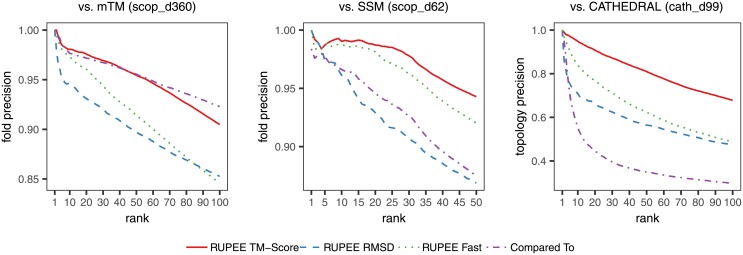
Precision for RUPEE fast, RUPEE top-aligned sorted by TM-score, and RUPEE top-aligned sorted by RMSD.

Fig 6 shows precision (i.e. positive predictive value or PPV) averaged over all searches, where positive results are defined as domains with the same classification for the indicated hierarchy level as the query domain. A plot of recall is unnecessary since Fig 6 provides precision at specific ranks for identical sets of searches. Hence, recall curves have the same relative relationships as those shown for precision.

We should expect a structure search to have reasonable precision with respect to the hierarchy levels of the structure classification it is searching. However, it is not clear how to define reasonable. On the other hand, if precision is too high, the search provides little value beyond that provided by the structure classification hierarchy it is searching. Towards the extreme end of high precision, it would be sufficient for a search to return the best match and from there refer to the hierarchy for additional results.

RUPEE fast and top-aligned sorted by TM-score show higher precision than SSM and CATHEDRAL. mTM shows higher precision than RUPEE fast. However, RUPEE top-aligned sorted by TM-score shows equal or higher precision than mTM up to the 50^th^ result and then drops below that of mTM. The lower precision after the 50^th^ result can be attributed, in large part, to the differences in normalization. A good example of this is for the search on domain d1j6va_ from scop_d360 having 148 residues. After the initial block of closely matched structures, mTM begins returning structures within the same fold with more than 200 residues like d1q2la2 and d1q2la3. On the other hand, RUPEE begins to look outside the fold to structures like d2fyxb2 and d2fyxa1 that provide better full-length alignments with respect to both the query and the target protein.

RUPEE top-aligned sorted by RMSD shows higher precision than for CATHEDRAL but lower precision than mTM and SSM. Also, RUPEE top-aligned sorted by RMSD shows lower precision than both RUPEE fast and RUPEE top-aligned sorted by TM-score. The lower precision for RUPEE top-aligned sorted by RMSD is a direct consequence of RMSD not being suitable for full-length alignments as discussed above in *Related work*. For this reason, it is advised to sort RUPEE top-aligned results by TM-score. RUPEE top-align provides a sort by RMSD only for the sake of completeness.

### Response times

Response times to a large degree are a measure of the amount of resources available to an application. Response times for RUPEE were gathered from the RUPEE web site running on a single Amazon Web Services (AWS) c5.2xlarge elastic compute (EC2) unit. With more resources, RUPEE response times can be further improved since pairwise alignments can be run in parallel. For mTM, SSM, and CATHEDRAL, we gathered response times by automating their respective web sites using the Selenium WebDriver API.

Given that our response time comparisons are made against the respective tools running in different environments, we cannot derive solid conclusions about the efficiency of the methods themselves. Nevertheless, these response times do fairly compare the user experience of the respective tools. Moreover, in some cases the response times differ dramatically, by an order of magnitude in the case of RUPEE fast.

**Fig 7:**
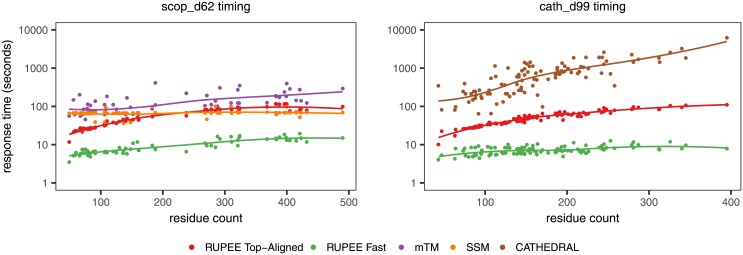
Response times for RUPEE fast and RUPEE top-aligned. The response times for RUPEE top-aligned are dominated by pairwise structure alignments and do not depend on the sort order.

Fig 7 shows response times in seconds for the scop_d62 and cath_d99 benchmarks. Here, we are able to show RUPEE fast and top-aligned, mTM and SSM on the same plot because scop_d62 is a subset of the scop_d360 benchmark. Both plots are shown with a logarithmic scale in order to include all outliers while still being able to view the overall trends in response times. Loess regression curves are also provided to further highlight the overall trends.

In all cases, RUPEE fast is considerably faster than all other searches. It is also clear that fast mode is not as sensitive to increasing residue counts in contrast to RUPEE top-aligned, mTM and CATHEDRAL. Response times for SSM are not affected by residue counts at all and always returns results in less than 100 seconds but this is at the expense of performance as shown in Fig 4.

The left plot of Fig 7 shows that RUPEE top-aligned is faster than mTM for all residue counts. Likewise, the right plot of Fig 7 shows that RUPEE top-aligned is significantly faster than CATHEDRAL for all residue counts.

The trend of increasing response times for RUPEE top-aligned is a direct result of the pairwise structure comparisons that are performed on the top 8000 results provided by RUPEE fast mode.

## Discussion

As shown above, a purely geometric big data approach to protein structure search can compete with the best available protein structure searches. Nonetheless, there remain avenues for further improvement and investigation.

While our min-hashing and LSH scheme does allow for some flexibility in the size of matched protein structures, it is not specifically designed for containment searches. However, the initial min-hashing and LSH search does operate fast enough, within seconds, that it does present the possibility of executing multiple searches within the context of a single query. With more resources, we can distribute min-hash and band data across multiple compute units, with each data set representing a subset of chopped structure representations. With a 75% overlap of these subsets, RUPEE searches effectively become containment searches.

We have tried the overlapping subset idea and have found it to be effective. However, on a single compute unit, the time required is more than we are willing to accept for this first iteration of RUPEE.

One possible drawback of RUPEE is that in both fast and top-aligned modes, it only returns the top 400 results. Again, with more resources, this number can be increased, but there still remains a need for some kind of cut-off. Nonetheless, in most search tools, having to look past the first few hundred results usually indicates an ineffective search strategy. To this end, RUPEE provides filters that can be used for traversing structure space more efficiently. For SCOPe, CATH, and ECOD the user can instruct the search to only return domains that differ from the query structure at a chosen hierarchy level classification. Additionally, for CATH the user can filter results based on hierarchy level representatives. Since RUPEE does not rely on sequence clusters or pre-calculated results, these kinds of filters are easy to implement, do not reduce the number of returned results, and allow for the discovery of unexpected structural similarities across classification hierarchies.

One area that stands out for possible improvement is our longest common subsequence (LCS) scoring adjustment to the results initially returned by RUPEE. While the LCS step has been shown to be effective, it is notable for its simplicity. A more complex step of validating the sequence of shingle matches can take a form similar to the path extension algorithm used by CE. In this case, sequences of matches would only be extended when the difference of interresidue distances between shingle pairs already in the sequence and a candidate pair to be added to the sequence fall below some threshold.

On the other hand, it would be interesting to see where further analysis of the initial results returned by RUPEE before LCS and any sort of order enforcement beyond that of the shingles themselves could lead. For instance, topological permutations such as circular permutations, segment-swapping and changing secondary structures within homologous proteins are not uncommon [37]. The initial RUPEE min-hashing and LSH algorithm provides candidate matches along with matched shingles. With some thought, an algorithm similar to FATCAT can be developed allowing for permutations in addition to twists.

## Conclusion

With the growth rate of solved structures deposited in the PDB, the need for a fast and scalable structure search is growing. Using run position encoded shingles of residue descriptors combined with min-hashing and LSH, we have shown that RUPEE fast is able to provide good results in seconds running on a single AWS elastic compute unit. Currently, RUPEE fast is the fastest available protein structure search providing the demonstrated level of accuracy. For RUPEE top-aligned, we have shown that a purely geometric big data approach to protein structure search is able to produce results equal to or better than the current state of the art protein structure searches that variously depend on clustered sequences or pre-calculated results. The ability for RUPEE to quickly examine all structures among hundreds of thousands sets it apart as a tool that can be used for discovering previously undetected structural similarities.

## Supporting information

S1 Benchmarks Domains included in benchmarks used for evaluation. (PDF)

S1 Fig Scoring from CE pairwise alignments. (PDF)

S2 Fig Scoring from FATCAT pairwise alignments. (PDF)

S1 Methods Run factors for shingles instead of descriptors. (PDF)
