# Supplementary material for: RUPEE: A fast and accurate purely geometric protein structure search
Source: PLoS One. 2019 Mar 15;14(3):e0213712. doi: 10.1371/journal.pone.0213712 (PMC6420038; doi:10.1371/journal.pone.0213712)
Supplement: S1 Benchmarks — (PDF) [file pone.0213712.s001.pdf]

1 Benchmarks

1.1 scop_d360

d1a1ca_,d1a92b_,d1ao6b1,d1b0ea_,d1bhaa_,d1bspa_,d1bwza1,d1bzqd_,d1c1ba2,d1c3pa_,
d1c81a_,d1cklf1,d1d5nc1,d1d6ha1,d1d8la1,d1de0a_,d1di0c_,d1djwb2,d1e0ca1,d1e9ha_,
d1egya_,d1ek1a2,d1f9qa_,d1ffxa_,d1fh9a_,d1fmta2,d1fyfa1,d1fyfa2,d1g3it_,d1g63i_,
d1gg3b2,d1gk8k_,d1gqme_,d1gtea4,d1gvra_,d1gztd_,d1hnvb_,d1hzyb_,d1i7pa2,d1ia2a_,
d1ii2a2,d1ik4f_,d1isba2,d1iwao1,d1j6va_,d1jdya3,d1jeqa1,d1jh2a_,d1jjid_,d1jrqb2,
d1jxja2,d1jyzm3,d1k9jb_,d1kkmb_,d1kkmj_,d1kooc1,d1kpsc_,d1kq4a1,d1krbc2,d1ksda1,
d1l6kj_,d1lcpa1,d1liwa6,d1lvha_,d1m80a3,d1mokc1,d1mpsh1,d1mwia_,d1ndqa_,d1ni6c_,
d1nkza_,d1nzwb_,d1o1cv_,d1odsf_,d1ogcd_,d1oh9a_,d1ojna2,d1p9ka1,d1perl_,d1pf9i3,
d1px3b4,d1q4qf_,d1q6za1,d1q86d_,d1qb8a_,d1qj3b_,d1qrqa_,d1qs7c_,d1qvrc3,d1r67a1,
d1roma_,d1s5ma_,d1sbka1,d1sg9a_,d1smyk2,d1srgb_,d1sw6b_,d1sxua_,d1szsa1,d1th8a1,
d1tjta2,d1tkab1,d1tkba3,d1tr9a1,d1ttob_,d1tyzd_,d1tzyd_,d1u9ma_,d1uc8a1,d1uf5a_,
d1uj1b_,d1uj4a1,d1upla_,d1v1tb1,d1v54u_,d1vq5d1,d1w7ab2,d1w7vd2,d1wpua_,d1x0sa_,
d1x1yd1,d1x92b1,d1xckd1,d1xmzb1,d1xn2c_,d1xnwe2,d1xuoa_,d1xvaa_,d1xyla_,d1y3be2,
d1y69k1,d1yf1c_,d1ygcl_,d1yhut_,d1yima_,d1yl4w1,d1yo6c_,d1yq2a4,d1ywhm3,d1z25a3,
d1z3qa_,d1zcfg_,d1zdja_,d1zqqa4,d220la_,d2avyh1,d2b66k2,d2b7aa_,d2b9nf1,d2b9no1,
d2bb3a1,d2bfea1,d2bm4a1,d2bq8x_,d2bsqc1,d2c64a2,d2c9lz1,d2ds0a_,d2dtyd_,d2dxia2,
d2e1va2,d2f3fa_,d2fdsa1,d2fk3c1,d2fugu1,d2g7ga1,d2gdub2,d2gjwd2,d2gmqa1,d2hbxb_,
d2hd1a_,d2hh2a_,d2hhhc2,d2idrb_,d2igad1,d2itjb_,d2iw6b1,d2j31a1,d2j62b2,d2juaa1,
d2lq7a1,d2noea1,d2nuwa_,d2o1ba_,d2ofea_,d2oqef1,d2pf2a2,d2plsf2,d2puga2,d2q7qh_,
d2qbij1,d2qbra_,d2qexc1,d2qp1s1,d2quea_,d2ripa1,d2rmaq_,d2uxcg1,d2uynb_,d2v4eg_,
d2v6ma2,d2v7xa2,d2vggb2,d2vv6d_,d2vy0b_,d2wipa1,d2wipc1,d2wogb_,d2x7yb_,d2yzdg1,
d2z0ac1,d2z27b_,d2z4ng1,d2zfzd_,d2zwib_,d3a5cc3,d3abmi_,d3aend_,d3ah3b_,d3anzi1,
d3aofb_,d3ayzb_,d3b6oa_,d3bc5a1,d3bp9f_,d3bwia_,d3bzub2,d3c6qb_,d3cjbg1,d3cojc2,
d3d5dp1,d3dh1b1,d3dllw1,d3dugf2,d3dyab_,d3e6kb_,d3ej8a_,d3f1sa_,d3f3yb_,d3flqa_,
d3foub_,d3fvlc_,d3g3cb1,d3guag1,d3gw1a_,d3gw9b_,d3h66b_,d3h90c2,d3hjjc_,d3hlib_,
d3hqpf2,d3ikab_,d3is3a_,d3k3ai_,d3k8ca_,d3kfab1,d3kpbd_,d3l7uc_,d3l7zd2,d3m5ka_,
d3mg8r_,d3n6fb_,d3ne4a_,d3nfdf_,d3nvya2,d3nyxa1,d3o92b_,d3ohuf_,d3om5a_,d3ozqa1,
d3pwsb2,d3q6ja3,d3q7jb1,d3qpkb3,d3qu1b_,d3r83a_,d3resb_,d3so1c_,d3tbla1,d3tcrb_,
d3ubnc_,d3ujeb1,d3umld_,d3uwlb_,d3uyta_,d3vdba5,d3w7ob_,d3wefb_,d3wrta_,d3wyla_,
d3ximd_,d3ziaf3,d4a1ua_,d4a75g1,d4au0a_,d4b1tb_,d4b7ba_,d4b7qd_,d4bpzb1,d4cqli_,
d4d2oa_,d4d8gb1,d4db5a_,d4e4nb_,d4f5lb1,d4fkwa_,d4frtb_,d4h0ta2,d4hhyd1,d4hx6a1,
d4i5bb1,d4ii4b1,d4isob_,d4iuaa1,d4ixzc_,d4j7xa_,d4jarc1,d4jcfa_,d4jhxc2,d4jyka2,
d4jyox_,d4k1wc2,d4k64c1,d4kv5a_,d4ltco_,d4lzwb_,d4mq6b_,d4mswc_,d4mv6a2,d4mw5b1,
d4napb_,d4nbjh_,d4nhzc2,d4otaa_,d4p3ya3,d4pd4f_,d4pitb1,d4pvma_,d4q1la_,d4q1sy_,
d4q2va_,d4qseb_,d4qv0c_,d4qv4o_,d4qv5e_,d4qv8l_,d4qzwy_,d4r17d_,d4ryva_,d4tvab4,
d4u39g1,d4unwa_,d4xheh1,d4xkdf_,d4yuya_,d4zgla_,d5acra_,d5cpya_,d5rlaa_,d7ccpa_

1.2 scop_d62

d1ao6b1,d1b0ea_,d1brwa1,d1bspa_,d1bzqd_,d1c1ba2,d1cklf1,d1d8la1,d1di0c_,d1dm5c_,
d1e9ha_,d1egya_,d1f9qa_,d1fyfa1,d1g3it_,d1gg3b2,d1gqme_,d1hc7c3,d1hnvb_,d1i6hf_,
d1ia2a_,d1isba2,d1iwao1,d1j6va_,d1jrqb2,d1jxja2,d1kkmj_,d1kpsc_,d1ndqa_,d1nzwb_,

d1o1cv_,d1ogbb1,d1pf9i3,d1px3b4,d1qj3b_,d1qrqa_,d1rhgc_,d1roma_,d1s5ma_,d1srgb_,
d1szsa1,d1tyzd_,d1tzyd_,d1v1tb1,d1v54u_,d1xyla_,d1zdja_,d1zqqa4,d1zr4e2,d220la_,
d2c64a2,d2f4vp1,d2igad1,d2iw6b1,d2nvqi2,d2oqef1,d2puga2,d2rmaq_,d3ximd_,d4otaa_,
d5rlaa_,d7ccpa_

1.3 cath_d99

1bxoA02,1byiA00,1hdoA00,1jfbA00,1k5nA01,1l3kA01,1lqtB02,1n3lA01,1n5uA03,1nkiA00,
1nwwA00,1nz0D00,1psrA00,1r0mA01,1rl6A02,1rtqA00,1swyA00,1unqA00,1vimC00,1wmwB00,
1xmkA00,2bw4A01,2dkjA02,2fvyA01,2nrlA00,2o9sA00,2ob3A00,2oizA01,2osxA01,2rbkA01,
2vb1A00,2vxnA00,2w8tA01,2wn9D00,2wurA00,2ylbC00,2zcmA00,3a02A00,3dlcA00,3e2oA01,
3go9A02,3goeA00,3h7iA02,3hj4A02,3iohA00,3kffA00,3ks3A00,3ku3A02,3ku3B00,3lqbA00,
3nbcA00,3sovA02,3t5tB01,3u7qA02,3uljB00,3w5hA01,3wh2A00,3ziyA03,4bj0A00,4cayB00,
4cbuA01,4cvrA00,4d3tA02,4d3tA03,4dd5A01,4ep4A00,4f1vA02,4fvyA01,4g1qA04,4k8gA02,
4l8aA00,4mf5A02,4mtuA00,4oh7A02,4pf3A00,4r2xD00,4ua6A00,4unuA00,4xemA01,4yapA01,
4z8jA00,4zflD00,5a71A00,5avdA02,5cgqB01,5cphA00,5dp2A01,5dzeA00,5hyvA02,5ibnA00,
5jbxB01,5jryA01,5jryA02,5k8sB00,5kvsA02,5lvoA01,5lvoA02,5m17A00,5sy4A00
