# Supplementary material for: RUPEE: A fast and accurate purely geometric protein structure search
Source: PLoS One. 2019 Mar 15;14(3):e0213712. doi: 10.1371/journal.pone.0213712 (PMC6420038; doi:10.1371/journal.pone.0213712)
Supplement: S2 Fig — (PDF) [file pone.0213712.s003.pdf]

1 Fig

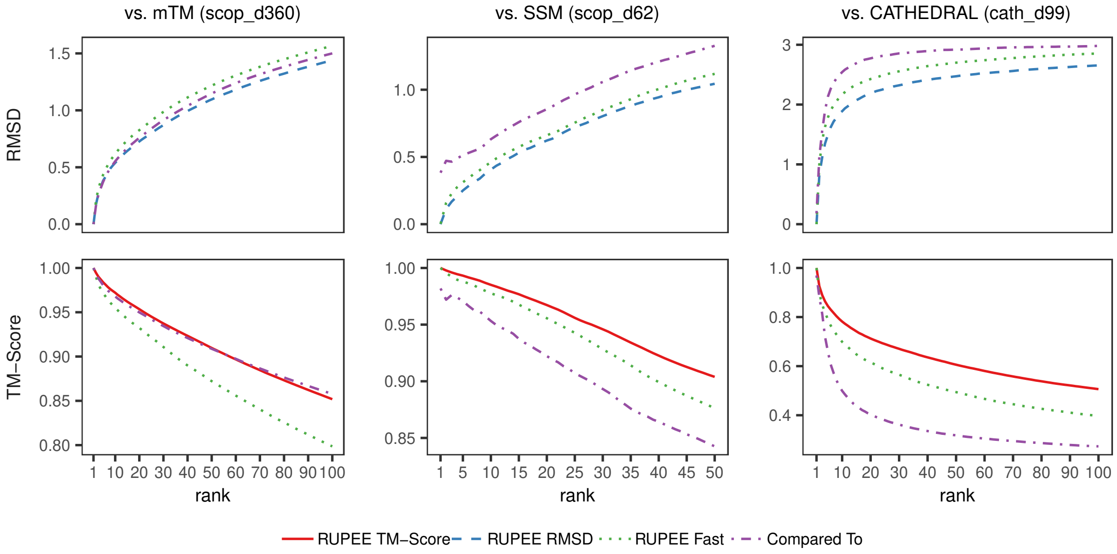

Figure 1: Scoring from FATCAT pairwise alignments for RUPEE fast, RUPEE top-aligned sorted by TM-Score, and RUPEE top-aligned sorted by RMSD
