# Supplementary material for: RUPEE: A fast and accurate purely geometric protein structure search
Source: PLoS One. 2019 Mar 15;14(3):e0213712. doi: 10.1371/journal.pone.0213712 (PMC6420038; doi:10.1371/journal.pone.0213712)
Supplement: S1 Methods — (PDF) [file pone.0213712.s004.pdf]

1 Methods

To see why RPE run factors are calculated at the descriptor level and factored in at the shingle level, consider shingling a list of RPE run factors themselves, which mirrors applying them at the descriptor level.

The sequence of RPE factors

$[\,0, 1, 2, 3, 2, 1, 0\,]$ becomes

$\{\,[0, 1, 2, 3], [1, 2, 3, 2], [2, 3, 2, 1], [3, 2, 1, 0]\,\}$

and with one less element

$[\,0, 1, 2, 2, 1, 0\,]$ becomes

$\{\,[0, 1, 2, 2], [1, 2, 2, 1], [2, 2, 1, 0]\,\}$

Notice above, there is not a single match for this one-off difference in run length. Now consider shingling a list of RPE factors, but this time all elements in the shingle are set equal to the first element in the shingle, which mirrors applying them at the shingle level.

The sequence of RPE factors

$[\,0, 1, 2, 3, 2, 1, 0\,]$ becomes

$\{\,[0, 0, 0, 0], [1, 1, 1, 1], [2, 2, 2, 2], [3, 3, 3, 3]\,\}$

and with one less element

$[\,0, 1, 2, 2, 1, 0\,]$ becomes

$\{\,[0, 0, 0, 0], [1, 1, 1, 1], [2, 2, 2, 2]\,\}$

In this latter case, a one-off difference in run length results in one less shingle match while still serving to increase the specificity of the shingles.
